# Sound waves alter the viability of tobacco cells via changes in cytosolic calcium, membrane integrity, and cell wall composition

**DOI:** 10.1371/journal.pone.0299055

**Published:** 2024-03-11

**Authors:** Mahsa Sardari, Faezeh Ghanati, Hamid Mobasheri, Abazar Hajnorouzi

**Affiliations:** 1 Department of Plant Biology, Faculty of Biological Science, Tarbiat Modares University, Tehran, Iran; 2 Laboratory of Membrane Biophysics and Macromolecules, Institute of Biochemistry and Biophysics, University of Tehran, Tehran, Iran; 3 Department of Physics, Faculty of Basic Sciences, Shahed University, Tehran, Iran; Universidad Autónoma Agraria Antonio Narro, MEXICO

## Abstract

The effect of sound waves (SWs) on plant cells can be considered as important as other mechanical stimuli like touch, wind, rain, and gravity, causing certain responses associated with the downstream signaling pathways on the whole plant. The objective of the present study was to elucidate the response of suspension-cultured tobacco cells (*Nicotiana tabacum* L. cv Burley 21) to SW at different intensities. The sinusoidal SW (1,000 Hz) was produced through a signal generator, amplified, and beamed to the one layer floating tobacco cells inside a soundproof chamber at intensities of 60, 75, and 90 dB at the plate level for 15, 30, 45, and 60 min. Calibration of the applied SW intensities, accuracy, and uniformity of SW was performed by a sound level meter, and the cells were treated. The effect of SW on tobacco cells was monitored by quantitation of cytosolic calcium, redox status, membrane integrity, wall components, and the activity of wall modifying enzymes. Cytosolic calcium ions increased as a function of sound intensity with a maximum level of 90 dB. Exposure to 90 dB was also accompanied by a significant increase of H_2_O_2_ and membrane lipid peroxidation rate but the reduction of total antioxidant and radical scavenging capacities. The increase of wall rigidity in these cells was attributed to an increase in wall-bound phenolic acids and lignin and the activities of phenylalanine ammonia-lyase and covalently bound peroxidase. In comparison, in 60- and 75 dB, radical scavenging capacity increased, and the activity of wall stiffening enzymes reduced, but cell viability showed no changes. The outcome of the current study reveals that the impact of SW on plant cells is started by an increase in cytosolic calcium. However, upon calcium signaling, downstream events, including alteration of H_2_O_2_ and cell redox status and the activities of wall modifying enzymes, determined the extent of SW effects on tobacco cells.

## Introduction

A sound wave (SW) is a vibrating and oscillating pressure that is transmitted through gas, liquid, and solid. It is an external mechanical force that, like other mechanical stimuli such as wind, rain, contact, and vibration, modulates the growth and development of living cells [1]. In case of attacks by pathogenic insects, some plants recognize and respond to the chewing sounds of insect larvae and also the noise of the pollinating bees, making them immune to their hazards [2]. The effects of sounds on different animal species, i.e., amphibians, arthropods, birds, fish mammals, mollusks, and reptilians, have been intensively investigated, and anthropogenic noise has been considered a serious form of environmental pollution [3].

Although the sound effect on plants has been an emerging area of research for the past few years and has fascinated the interests of investigations, the mechanism(s) by which sound influences plant growth and development have not been widely elucidated [1]. Nonetheless, it has been shown that plants can perceive external sound effects, and the existence of complex molecular mechanism(s) for SW perception and signal transduction has been suggested [4]. It has been observed that some plants can produce sounds and transmit information through the xylem tissue [5].

Similar to other mechanical forces, e.g., gravity, touch, and hyper osmosis, SW in plants is sensed by membrane-bound mechanosensitive channels, mainly MSLs (the non-selective mechano-sensitive-like) and Calcium-specific (Ca^2+^-specific) channels (MCA) [2, 6]. Some mechanosensitive ion channels, MSL9 and MSL10, have been recognized in the plasma membrane of root cells of *Arabidopsis thaliana* [7]. The mechanosensitive Ca^2+^-dependent NtMCA1 and NtMCA2 channels in the plasma membrane of tobacco BY-2 cells regulate the Ca^2+^ influx through the plasma membrane and are involved in the proliferation and gene expression induced by mechanical stress [8].

The expression of *A*. *thaliana* MCA1 in xenopus oocytes enhances the mechano-sensitive channel activity, suggesting that plants’ MCA can also respond to mechanical stimuli as well as MSLs [2]. It has been suggested that upon perception of SW by mechanical receptors, the increase in the level of intracellular Ca^2+^, which is followed by signaling pathways, is mediated by certain proteins in the wall-membrane complex and ultimately changes the arrangement and activity of cellular microtubules [2]. Nevertheless, there is a large gap in understanding SW-mediated molecular changes [1, 9]. Ca^2+^ signatures initiated from sound sensing can cause extensive changes in the levels of gene transcription through calcineurin B-like proteins (CBLs) and CBL-interacting protein kinases (CIPKs), thereby regulating production and activities of radical scavengers, enzymatic and structural proteins, and hormones. Regulation of the expression of several touch-responsive genes after exposure to acoustic waves has also been reported, suggesting possible molecular crosstalk occurring between different mechanical stimuli [10–12].

SW can also regulate the growth, phytochemical contents, and stress responses of plants and thus have the potential to increase the quality of plant products and strengthen the plant’s immunity against pathogens [13]. An increase in the glucosinolate and anthocyanin contents, modulation of stress-related gene expression, and resistance to *Botrytis cinerea* were observed in *A*. *thaliana* in response to the vibrations produced by insect herbivores while chewing on leaves [14]. Stimulation of strawberry fruits with sounds at 1,000 Hz and 100 dB increased several fruit quality parameters and enhanced defensive metabolites [15]. Also, exposure of *Chrysanthemum* calli to the sound of 1,000 Hz and 100 dB increased the activity of plasma membrane H^+^-ATPase via Ca^2+^-dependent phosphorylation [16, 17]. The tobacco cell walls consist of polysaccharides, structural proteins, enzymes, aromatic substances, and other molecules that fabricate certain expandable and deformative architecture. Due to the inter- and intra-molecular interactions between cell wall constituents, they possess unique dynamics required to undergo various physiological functions, including defense against pathogens, signal transduction, nutrient uptake, and so on [18]. Consequently, exposure of tobacco cells to mechanical SW at a certain frequency and intensity/pressure may alter their dynamics, deviating from the normal dynamic and, thus, affecting the function of the channels, receptors, and ultimate physiology of the cells [19].

SW is typically characterized by its frequency (Hertz) and intensity (dB), a logarithmic unit to measure sound level representing sound pressure [20]. Sound waves are categorized based on their frequency into infrasound (≤ 20Hz), ultrasound (≥ 20 KHz), and audible waves (20 Hz to 20 kHz) groups [20]. Normal conversational speech strength is about 60 dB (moderate); in chamber music, the small auditorium is about 75 dB (loud) and about 90 dB (very loud) for the train whistle at 500 feet [21, 22]. We can consider a sound level of 0 dB as a reference level of sound that equals a pressure of 20 μPa, a pressure that a normal, healthy young ear can sense. Doubling sound pressure (in Pa or N/m^2^) results in an increase in sound pressure level (in dB) by 6 dB (or 20 log 2) [23].

To the best of our knowledge, there are a few experiments have been conducted on individual plant cells to address the sound induced pressure on the cell wall integrity, lipid peroxidation, free radical and H_2_O_2_ production, cytosolic free and total calcium levels, and enzyme activities in tobacco cells so far [24, 25]. The present biophysical study was conducted to monitor the innate physiological responses of suspension cultures of tobacco cells to the applied SW. Considering the presence of Ca^2+^-dependent ion channels in the membrane of the treated cells, it seems that they have been directly affected by the mechanical pressure induced by SW, resulting in the increase of the Ca^2+^ concentration, thereby triggering downstream dominos that led to the above physiological changes.

Accordingly, we believe the current approach paves the path for further studies and application of SW as an alternative means to chemical treatments to increase the resistance of plants to parasites and improve their viability and growth in a non-chemical, cheap, fast, and effective manner.

## Material and method

### Tobacco cell culture and growth characteristics

All methods were carried out in accordance with the relevant national guidelines and legislation. According to the Research Ethics Committee of Tarbiat Modares University, no certificate was needed for this research.

A suspension-cultured line of tobacco (*Nicotiana tabacum* L. cv Burley 21) grown in a modified LS medium was used [26]. The cells were grown on reciprocal shakers (123 rpm) in a room with a SW level of 55 ± 2 dB, 25± 2°C. The sound generator and amplifier were put out of the chamber to avoid mechanical vibrations. The cells in the middle of the logarithmic growth phase (day 7) were transferred to a locally designed sound-proof chamber. The experiment was accomplished early morning, in a quiet place. The lack of any noise and environmental sound inside the chamber was tested by a sound meter and no significant external sound wave was recorded when the defined source was off.

**Fig 1:**
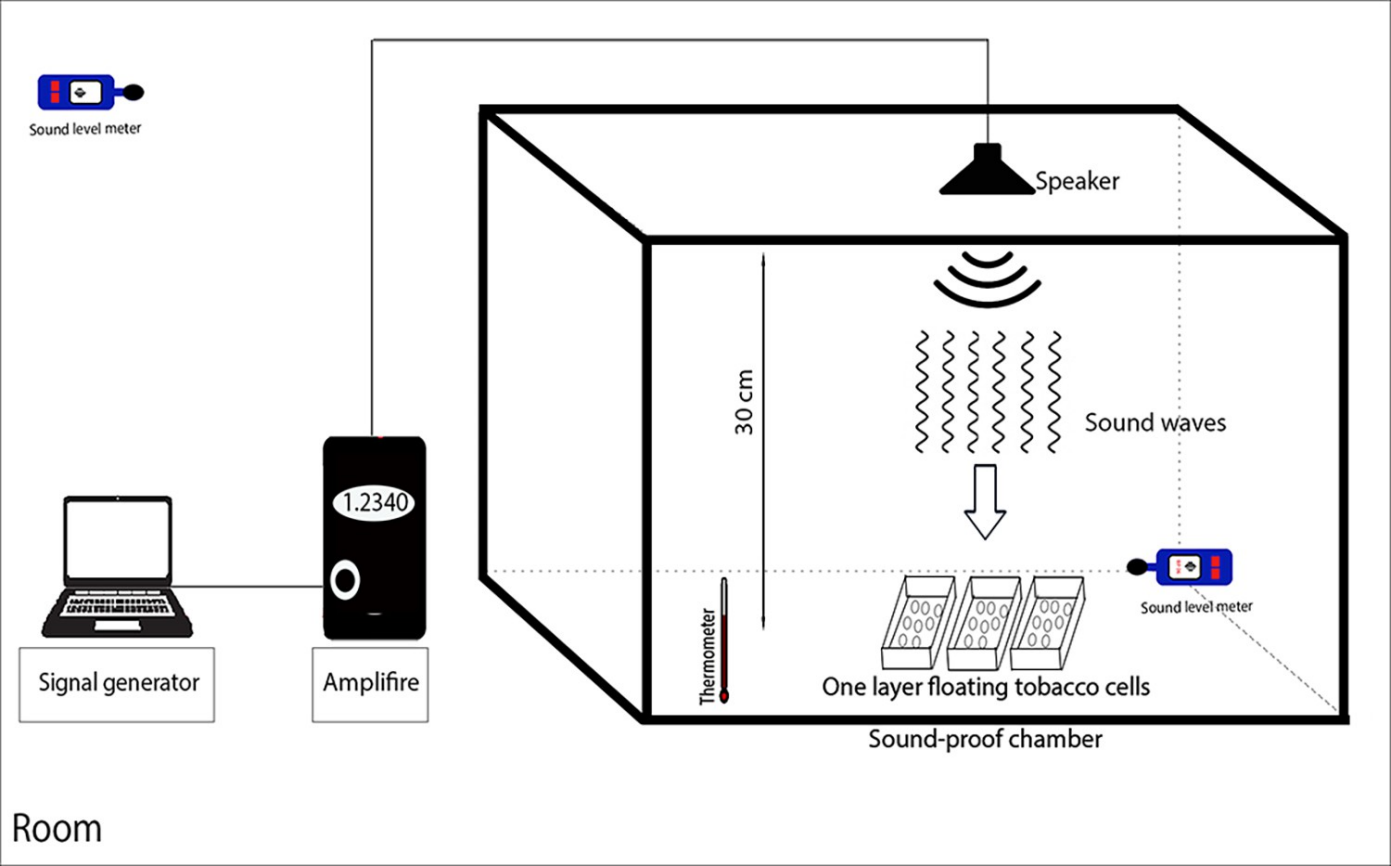
Schematic diagram of the sound wave applying setup.

The cells were then floated on a medium containing rectangular plates (20 × 40 cm) and exposed to sinusoidal SW with intensities of 60, 75, and 90 dB at a constant frequency of 1000 Hz for 15, 30, 45, and 60 min (Fig 1). The stimulation frequency of 1,000 Hz was chosen based on previous studies [27–29]. At the time of treatment, the control cells were placed in similar conditions in a sound-proof chamber out of a shaker at the same temperature and humidity. After treatment, they were transferred to the growth room for 24 h and then harvested under reduced pressure. Some of the aliquots of the cells were used to measure their viability, and the others were frozen in liquid nitrogen and kept in a freezer at -80°C for further analysis.

The viability of the cells was determined using Evans’ blue dye [26]. The fresh and dry weights of the cells (FW and DW, respectively) and total protein content were used as the growth indexes. Bradford assay relies on the absorbance maximum shift of the coomassie brilliant blue G-250 dye from 465 to 595 nm after binding to denatured proteins in solution and was used to determine the concentration of protein [30].

### SW application and its corresponding pressure imposed on live tobacco cells

The SW was generated by Adobe Audition version 3.0 software (USA). The intensity and uniformity of SW were measured during the treatment period at the level of cell-containing plates by a sound level meter (Casella, CEL490, UK). The pressure imposed by SW was worked out according to Eq 1:

Lp=20log(P/Pref) Eq (1)

where Lp is the sound pressure level (dB), P is the measured sound pressure, and Pref is the 2.10^−5^ reference pressure (Pa). Accordingly, the pressures of the SW 60, 75, and 90 dB were equal to 20, 112.5, and 632.5 mPa or mN/m^2^, respectively.

### Biochemical and biophysical analysis of tobacco cells in culture

In order to measure the total Ca^2+^ content of the cell samples, they were ashed at 350°C (2 h) and 550°C (3 h), digested in 1 M HCl: deionized water (1:1) solution, evaporated in a sand bath (110°C) and extracted in 1 M HCl, eventually. The total Ca^2+^ content was measured by an Atomic Absorption instrument (Shimadzu, AA-670, Japan). The concentration of cytosolic free Ca^2+^ was determined by loading cells with the acetoxymethyl ester of the Ca^2+^-binding dye Fura-2 (Fura-2-AM, Molecular Probes, Sigma-Aldrich, USA) in the dark at 28°C for 1 h. The cells were then washed three times with fresh medium and left for 15 min to have the Fura-2 crossed cell membranes and entered the cells where the cytosolic esterases cleave the acetoxymethyl hydrophobic side chains and produce the hydrophilic permeable fluorescent dye/ Ca^2+^ complex. Ca^2+^-bound Fura-2 AM had an excitation maximum of 340 nm, while the Ca^2+^-free Fura-2 AM maximum excitation happened at 380 nm. In both states, the emission maximum was about 510 nm [31].

Cellulase activity was measured using 3,5-dinitro salicylic acid (DNS). In this assay, cellulase breaks cellulose into glucose, and then the carbonyl group of glucose is oxidized to aldehyde, meanwhile, DNS is reduced to 3-amino-5-nitrosalicylic acid [32]. The activity of endoglucanase (EGase) was determined based on the release of glucose from carboxymethyl cellulose. The released glucose was measured by the phenol sulfuric method [33]. In both cases, one unit (U) of enzyme activity was defined as the concentration (μM) of released glucose per min [34]. The activity of phenylalanine ammonia lyase (PAL), the key enzyme in the metabolism of phenolic compounds, was measured based on the known mechanism of conversion of phenylalanine to trans-cinnamic acid during 60 min treatment [35]. The activity of peroxidase was measured using guaiacol and syringaldazin substrates in soluble, ionically, and covalently wall-bound fractions defined as SPO, IPO, and CPO, respectively [26].

The cell wall was isolated using EtOH, a mixture of CHCl_3_: MeOH (1:1 v/v), and acetone, followed by filtration, and dried eventually. Pectin was extracted from the resulting dried wall powder, stepwise, solubilized in hot ammonium oxalate (20 mM, 70°C), and then NaOH 0.1 M, freeze dried, and then weighed. A dissolving solution containing Sodium hydroxide (17.5%) and sodium borohydride (0.02%) was then used to extract hemicellulose. The presence of HOAc precipitated hemicellulose A (HA), and the supernatant was freeze dried and defined as hemicellulose B (HB). After washing the pellet with a mixture of EtOH and Et_2_O (1:1), the resulting cellulose was dried and weighed [36].

Major wall-bound phenolic acids i.e., cinnamic acid, caffeic acids, *p*-coumaric, and ferulic acid were extracted from pectin by EtOAc, air-dried, re-dissolved in 50% MeOH, and determined by HPLC (Waters, e2695, USA) equipped with C18 column (Perfectsil Target ODS3, 5 μm, 250 × 4.6 mm, MZ-Analysentechnik, Mainz, Germany). Phenolics were eluted at a flow rate of 0.5 mL min^-1^ with a linear gradient of 30–80% MeOH containing 0.1% HOAc and were detected at 280 nm using a 2489 UV-vis detector [37].

The Lignin content was determined in fine powdered, air-dried cell wall polysaccharide (CWP) by a modified acetyl bromide procedure. In brief, the powder was suspended in a mixture of AcBr (25%, w/w) in HOAc and HClO_4_ (70%) shaken at 10 min intervals at 70°C for 30 min. After cooling with ice, the digestion mixture was added to the NaOH (2 N) solution, and HOAc was added again. The Lignin content was determined by measuring the absorbance at 280 nm using a UV-visible double-beam spectrophotometer (GBC-Cintra6, Australia). A specific absorption coefficient value of 20 g L^-1^ cm^-1^ was used to calculate lignin content [36]. The total content of soluble phenolics was measured in methanolic extract of the cells using the Folin-Ciocalteu reagent [38]. For quantitation of H_2_O_2_, the samples were extracted with 0.1% trichloroacetic acid (TCA) and then KI was added. In this acidic condition, KI was dissociated to iodide ions which reacted with H_2_O_2_ and produced iodine. The latter was detected at 390 nm [39].

The rate of peroxidation of membrane lipids was assessed by measuring the concentration of malondialdehyde (MDA), which is the end product of peroxidation of membrane lipids [40]. The ferric ion reducing antioxidant power (FRAP) of the samples was determined based on the oxidation-reduction of potassium ferricyanide and ferric chloride in the presence of TCA, using ascorbic acid as a positive control [41]. Total radical scavenging capacity (RSC) was determined using the stable 2,2’diphenyl picrylhydrazyl radical (DPPH) free radical [40].

### Statistical analysis

The experiments were conducted based on a completely randomized design (CRD) with three independent repetitions. ANOVA procedure of the statistical program SPSS (version 22, USA) was conducted for the analysis of variance. The differences between treated samples were evaluated using Duncan’s test and considered significant when *p* ≤ 0.05. The overall coefficients of variation (OCV) were calculated as the ratio of standard deviation to average ×100 using the average values of obtained experimental data. The minimum OCV values were subtracted from the maximum OCV values divided by 3 to work out the classification of OCV. Accordingly, the OCVs were classified as high (63.95 to 95.7%), medium (32.2 to 63.95%), and low (0.45 to 32.2%). Principal component analysis (PCA) was also performed by XLSTAT (version 2.14.14, 2023) to work out the association and correlation between variables and observed values.

## Results

### Effect of SW on the total and cytosolic free Ca^2+^ ions in tobacco cells

**Fig 2:**
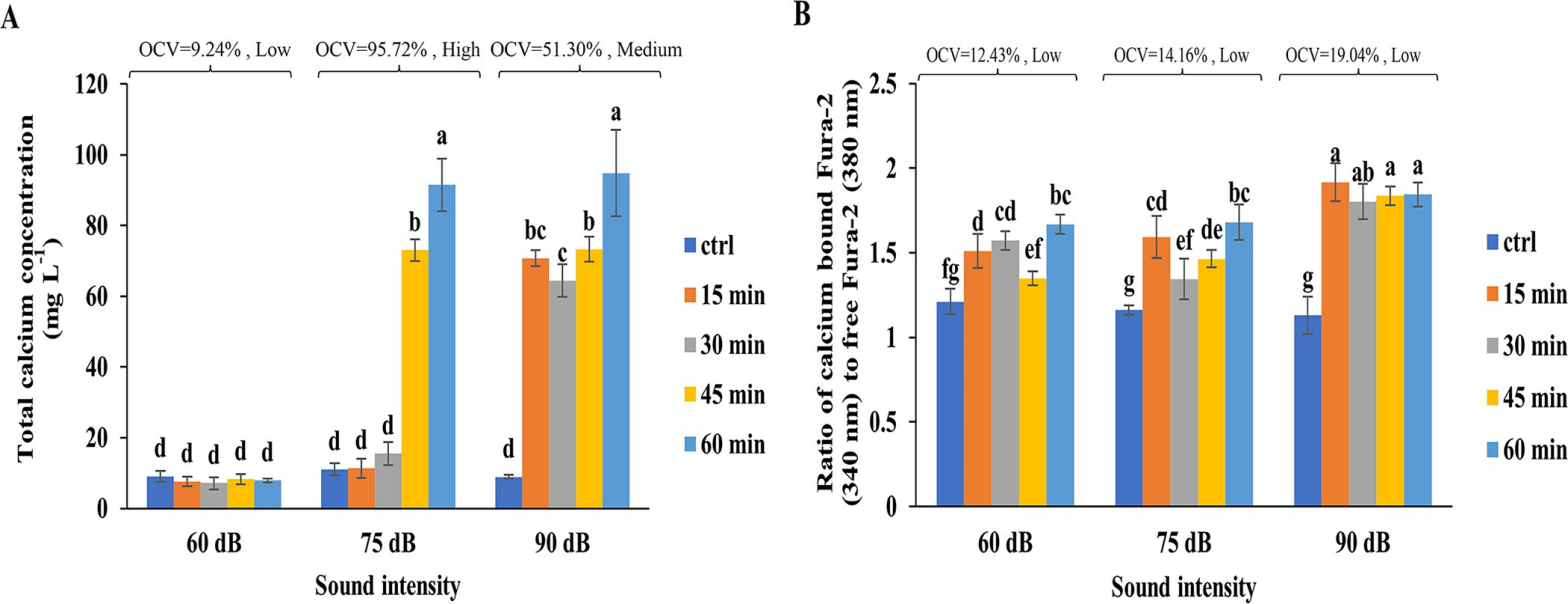
The effects of SW at different intensities and time lengths on the total and free calcium concentrations in tobacco cells. The total Ca^2+^ concentration of untreated (control) and treated tobacco cells with sound pressures of 60, 75, and 90 dB for 15,30,45, and 60 min are shown **(a)**. The increase of cytosolic Ca^2+^ was determined by the ratio of Ca^2+^-bound Fura-2 to Ca^2+^-free Fura-2 (340/380 nm) **(b)**. Different letters denoted on bars indicate significant differences (Duncan test, *p* ≤ 0.05). The overall coefficient of variation (OCV) was classified as low when it varied from 0.45 to 32.2%, medium from 32.2 to 63.95%, and high from 63.95 to 95.7%.

Exposure of cells to SW showed a significant effect on the total and cytosolic free Ca^2+^ as a function of dBs and exposure time length. The highest total calcium contents of the cells were detected at 75 dB and 90 dB after 60 min exposure to SW, almost 11 and 11.5 fold higher than that of the control, respectively (Fig 2A). The increase started as early as 15 min exposure at 90 dB but after 45 min at 75 dB. The OCV was low (9.24%) at 60 dB, medium (51.30%) at 90 dB, and high (95.72%) at 75 dB (Fig 2A). There were no changes identified at SW 60 dB during the course of the experiment and up to min 45 at 75 dB. The excitation spectrum was recorded for samples, and the ratios at 340 nm over 380 nm were plotted. As shown in Fig 2B with increasing sound intensities, a significant shift has been observed in λ_max_ of Fura-2-AM from 340 to 380 nm, indicating an increase in the cytosolic free Ca^2+^ concentration as a function of SW intensity. However, they showed low OCVs (Fig 2B). The maximum ratio of free Ca^2+^ to total ones as a result of exposure to SW was monitored when SW 90 dB was applied, and to some extent, the ratio correlated to the dB intensity, i.e., the higher the dB, the higher was the ratio.

### Effects of SW on the activity of the enzymes involved in phenolic metabolism in tobacco cells

**Fig 3:**
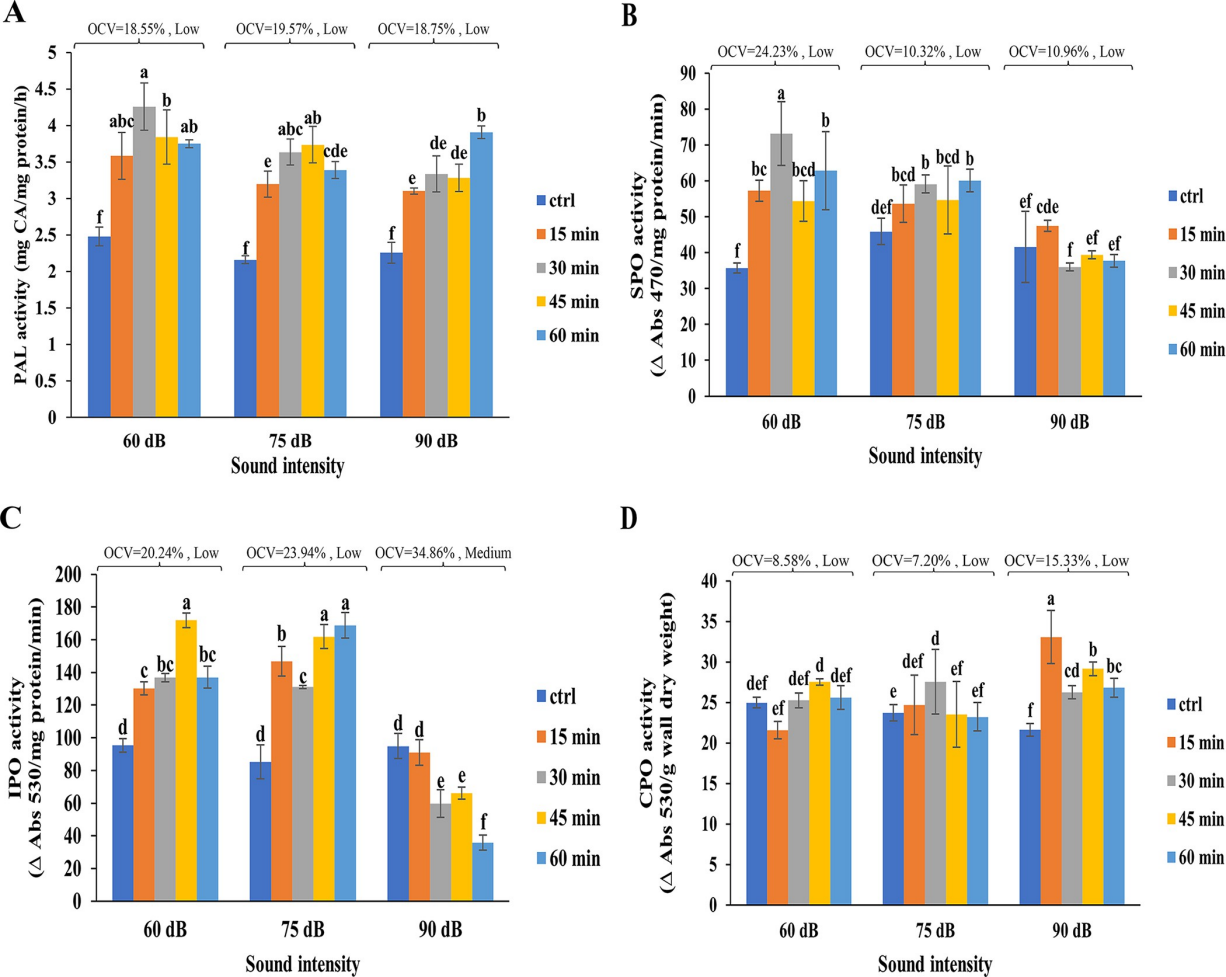
Effects of SW on the activity of the enzymes involved in phenolics metabolism in tobacco cells at different intensities and time lengths. The effect of SW 60, 75, and 90 dB on the activities of PAL **(a)**, SPO **(b)**, IPO **(C),** and CPO **(d)** applied for 15, 30, 45, and 60 min are shown. Different letters denoted on bars indicate significant differences (Duncan test, *p* ≤ 0.05). OCV was classified as low when it varied from 0.45 to 32.2%, medium from 32.2 to 63.95%, and high from 63.95 to 95.7%.

The activity of PAL in all SW treated cells was significantly higher (114% - 175%) than that of controls (Fig 3A). Furthermore, the activities of soluble and ionically wall-bound peroxidase increased after exposure to SW 60 dB (Fig 3B and 3C). However, no significant alteration was observed in the CPO activity as a result of exposure of cells to SW at 60, 75, and 90 dB. (Fig 3D). The trend of activity of peroxidase fractions in SW 75 dB-treated cells was identical to that of 60 dB (Fig 3B–3D). A significant decrease in IPO and an increase in CPO activities were identified in the cells treated with SW 90 dB, while the SPO activity remained unchanged. (Fig 3B–3D). Based on statistical analyses, OCVs of PAL, SPO, and CPO in all SW intensities were classified as low, while IPO at 90 dB was classified as medium (34.86%) (Fig 3A–3D).

### Effect of SW on the biochemical composition and characteristics of tobacco cell wall

The total content of the cell wall and its components are shown in Table 1. Exposure to SW at 60- and 75 dB for 30, 45, and 60 min significantly increased the CWP of tobacco cells. However, treatment of the cells at 90 dB had no significant effect on CWP (Table 1). The pectin content of the cells was remarkably reduced by 68%, 64%, and 41% when they were exposed to SW at intensities of 60, 75 and 90 dB, respectively (Table 1). A similar reducing trend was observed in the HB content (Table 1). However, the HA level increased by two folds when the cells were exposed to 60- and 75 dB (Table 1). Exposing cells to SW 60 dB increased their cellulose content by almost 300%, while at 90 dB, it was reduced by 45% (Table 1). The fluctuation of cellulose content in the 75 dB treated cells was a linear function of their SW treatment time length (Table 1).

**Table 1:** Alteration of cell wall components and soluble phenolics of suspension-cultured tobacco cells before and after exposure to sound waves.

Sound intensity (dB)	Exposure time (min)	CWP (mg g FW^-1^)	Pectin (mg g FW^-1^)	Hemicellulose A (mg g FW^-1^)	Hemicellulose B (mg g FW^-1^)	Cellulose (mg g FW^-1^)	Cinnamic acid (μ g FW^-1^)	*P*-Coumaric acid (μg g FW^-1^)	Caffeic acid (μg g FW^-1^)	Ferulic acid (μg g FW^-1^)	Total wall phenolics (μg g FW^-1^)	Lignin (mg g FW^-1^)		Soluble Phenolics (mg g FW^-1^)
60 dB	0 (Ctrl)	30.86±3.4^i^	4.9±0.4^bc^ (16)	4.7±1.2^efg^ (15)	1±0.1^ab^ (3)	6.3±0.1^k^ (20)	20±1.6^h^	5.3±0.5^a^	6.6±0.5^de^	3.6±0.0^bc^	36.6±1.6^i^	6.8±0.4^ef^	0.9±0.04^g^	
	15	32.30±0.7^hi^	3.7±0.3^efgh^ (12)	8.3±1.7^d^ (26)	0.9±0.1^bc^ (3)	8.1±0.4^ij^ (25)	38.3±0.9^d^	4.5±0.6^ab^	11.8±0.4^c^	3.5±0.0^c^	58.3±0.1^d^	7.2±0.2^de^	1.5±0.1^cd^	
	30	59.76±3.4^cd^	4.3±0.4^cde^ (7)	22±2.5^b^ (37)	0.7±0.1^cde^ (1)	15.3±0.7^ef^ (26)	41.4±1.2^c^	3±0.7^cd^	13.6±0.8^b^	3.6±0.1^bc^	61.8±0.6^c^	7.4±0.4^cd^	1.4±0.03^d^	
	45	58.96±2.4^cd^	3.8±0.3^efgh^ (6)	17.1±1.5^c^ (29)	0.6±0.1^ef^ (1)	23.7±0.3^c^ (40)	57.8±0.5^a^	4.3±1.1^abc^	13.8±0.6^b^	3.9±0.5^abc^	80.2±1.2^a^	7.4±0.1^cd^	1.5±0.1^abc^	
	60	73.01±3.2^b^	3.4±0.3^ghi^ (5)	21±1.8^b^ (29)	0.6±0.1^de^ (1)	29.4±1^b^ (40)	46.5±1.2^b^	2.4±0.6^d^	16.7±0.9^a^	3.6±0.1^bc^	69.4±0.7^b^	6.8±0.2^ef^	1.6±0.03^a^	
OCV (%)		36.43	14.65	52.94	23.9	59.95	33.80	30.17	29.89	4.17	26.34	4.26	20.11	
Classification of OCV*		Medium	Low	Medium	Low	Medium	Medium	Low	Low	Low	Low	Low	Low	
75 dB	0 (Ctrl)	36.46±0.8^gh^	5.2±0.5^b^ (14)	7.3±1.6^de^ (20)	1±0.1^ab^ (3)	8.6±0.6^hi^ (23)	33.5±1.5^f^	5.1±0.6^a^	7±0.6^de^	3.6±0.0^bc^	49.7±1.5^fg^	6.6±0.1^f^	0.8±0.03^h^	
	15	53.80±1.6^e^	3.5±0.4^fghi^ (7)	24±1.6^ab^ (45)	0.5±0.1^efg^ (1)	14.5±1^f^ (27)	41.3±0.8^c^	2.7±0.3^d^	6.9±0.8^de^	3.5±0.0^c^	54.7±0.2^e^	6.5±0.2^f^	1.1±0.1^f^	
	30	81.30±1.1^a^	2.8±0.1^i^ (3)	27±2.6^a^ (33)	0.8±0.1^bcd^ (1)	34.7±0.9^a^ (43)	36.1±1.3^e^	3±0.9bc^d^	7.8±0.9^d^	3.6±0.1b^c^	50.7±2.4^f^	7.4±0.1^cd^	1±0.1^f^	
	45	60.90±2.8^cd^	2.9±0.3^hi^ (5)	25±1.9^a^ (41)	0.3±0.1^gh^ (0.5)	16.6±0.7^e^ (27)	48±1.7^b^	1.7±0.2^d^	7.8±0.4^d^	3.5±0.0^c^	61.2±1.3^c^	7.1±0.1^de^	1.1±01^f^	
	60	60.20±2.5^cd^	3.1±0.4^hi^ (5)	27 ±1.5^a^ (45)	0.3±0.2^h^ (0.5)	11.3±0.5^g^ (19)	38±1.5^de^	1.9±0.4^d^	5±0.8^fg^	4.5±0.0^a^	49.7±0.8^fg^	7.6±0.1^bc^	1.3±0.04^e^	
OCV (%)		27.49	28.21	38.06	53.69	59.97	14.2	46.99	16.58	11.43	9.25	6.85	17.13	
Classification of OCV*		Low	Low	Medium	Medium	Medium	Low	Medium	Low	Low	Low	Low	Low	
90 dB	0 (Ctrl)	35.60±0.7^gh^	5.9±0.4^a^ (17)	7.2±1.3^def^ (20)	1.1±0.1^a^ (3)	7.1±0.7^jk^ (20)	26.1±0.6^g^	5±0.3^a^	6±0.7^ef^	3.5±0.0^c^	41.6±1.4^h^	6.8±0.1^ef^	0.9±0.1^fg^	
	15	45.33±2.1^g^	4±0.3^defg^ (9)	4.1±1.4^efg^ (9)	0.6±0.1^de^ (1.3)	18.4±0.4^d^ (40)	46.4±0.8^b^	2.5±0.8^d^	4.5±0.6^g^	4.2±0.6ab^c^	57.7±2^d^	6.8±0.1^ef^	1.6±0.04^bc^	
	30	49.56±2.3^f^	4.2±0.1^cdef^ (9)	4.9±0.1^efg^ (10)	0.7±0.1^cde^ (1.4)	16.5±0.9^e^ (33)	37.2±0.7^de^	3.2±0.2^bcd^	4.6±0.5^g^	4.5±0.1^a^	49.7±1.3^fg^	7.9±0.1^b^	1.4±0.1^cd^	
	45	33.76±2.5^hi^	4.3±0.4^cde^ (13)	3.8±0.8^fg^ (11)	0.4±0.1^fgh^ (1.1)	9.1±0.2^h^ (27)	38.7±0.8^d^	3.1±0.5^bcd^	7.2±0.7^de^	4.5±0.1^a^	53.6±2.5^e^	8.4±0.2^a^	1.6±0.1^ab^	
	60	46.43±3.3^fg^	4.6±0.2^bcd^ (10)	1.8±0.2^g^ (4)	0.4±0.1^fgh^ (1)	4.3±0.8^l^ (9)	33.5±0.7^f^	2.8±0.9^cd^	6.2±0.9^ef^	4.5±0.1^a^	47.1±0.4^g^	8.5±0.1^a^	1.2±0.1^e^	
OCV (%)		16.63	16.48	44.84	45.01	55.02	20.04	29.46	20.07	10.22	12.31	10.87	22.13	
Classification of OCV*		Low	Low	Medium	Medium	Medium	Low	Low	Low	Low	Low	Low	Low	

Different letters indicate significant differences by the Duncan test (*p* ≤ 0.05).

*Overall coefficients of variation (OCV) were classified as low when varied from 0.45 to 32.2%, medium from 32.2 to 63.95%, and high from 63.95 to 95.7%.

The content of major wall-bound phenolic acids, lignin, and the total content of soluble phenolics before and after exposure of tobacco cells to SW are shown in Table 1. In comparison with the control group, treatment of the cells with SW 60 dB significantly increased the content of both cinnamic acid (up to 2.9 of the control) and caffeic acids (2.5 fold of the control), while reduced *p*-coumaric acid and left that of ferulic acids unchanged (Table 1). The subsequent treatment with SW 75 dB increased cinnamic acid and decreased *p*-coumaric acid. When the exposure time increased to 60 min, the caffeic acid content of cell walls decreased to 70% of control, while ferulic acid increased significantly (Table 1). In SW 90 dB treated cells, the contents of cinnamic- and ferulic acids were significantly increased, whereas no significant changes in the caffeic acid content were identified (Table 1). The amount of *p*-coumaric acid reduced to 56% of control when cells were treated with SW 90 dB treatment, similar to its trend at SW 60 and 75 dB (Table 1). In comparison with controls, the lignin content of tobacco cells significantly increased after exposure to SW, particularly at treatment times of longer than 15 min (Table 1). The total concentration of soluble phenolics in all SW treated cells was higher than that of their corresponding controls (Table 1). At 60 dB treatments, OCVs of CWP, HA, cellulose, and cinnamic acid were medium and for other parameters were low. The OCVs calculated in 75 dB treated cells for HA, HB, cellulose, and *p*-coumaric acid were at medium levels. At 90 dB, OCV was medium for HA, HB, and cellulose and low for other parameters (Table 1).

**Fig 4:**
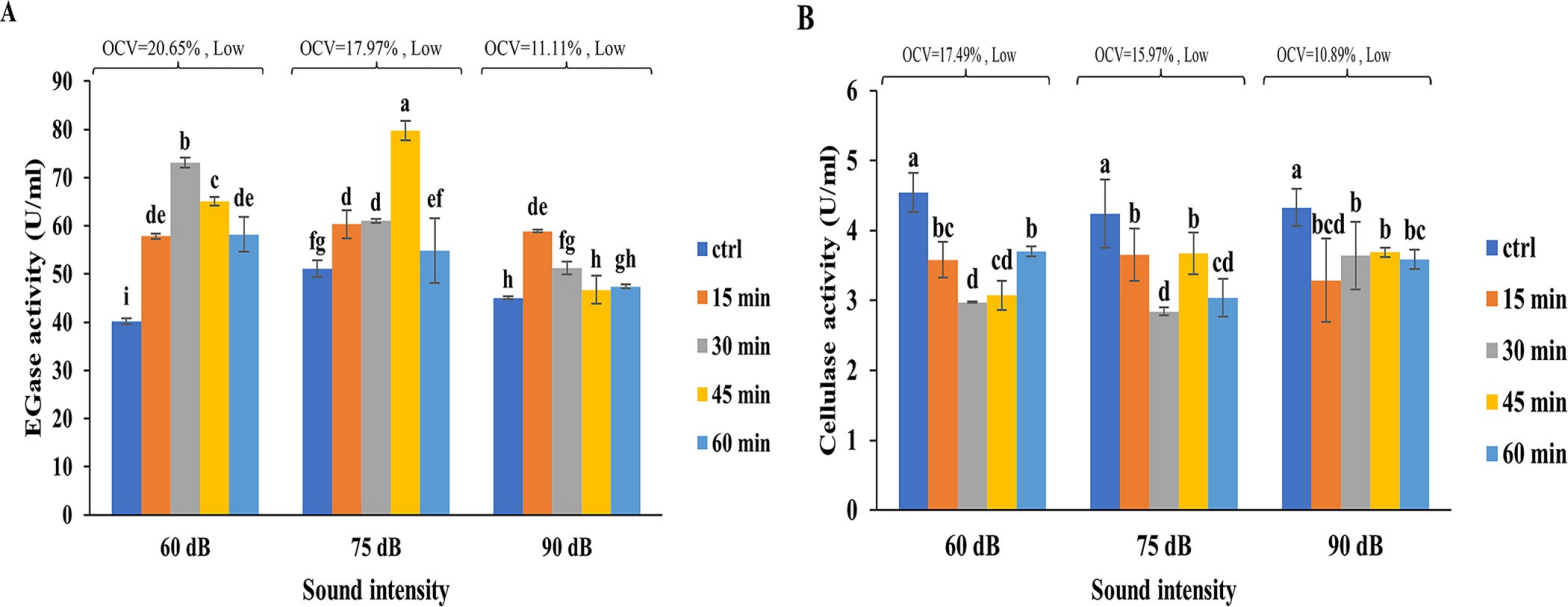
EGase and cellulase activities in cultured tobacco cells in response to SW at different intensities and exposure times. Activities of EGase **(a)** and cellulase **(b)** before and after exposure to SW of 60, 75, and 90 dB, each applied for 15, 30, 45, and 60 min, are shown. Different letters denoted on bars indicate significant differences (Duncan test, *p* ≤ 0.05). OCV was classified as low when it varied from 0.45 to 32.2%, medium from 32.2 to 63.95%, and high from 63.95 to 95.7%.

The activity of EGase in 60 and 75 dB treated cells up to 45 min of exposure time was ca. 1.2–1.5 folds higher than that of controls (Fig 4A). Exposure of 90 dB treated cells to SW, however, caused an increase in the EGase activity only after 15 and 30 min and returned to the control level after 45 and 60 min (Fig 4A). The activity of cellulase was significantly lowered in all SW treated cells irrespective of the length of treatments (Fig 4B). Low OCVs were noted for EGase and cellulase under all SW exposure conditions.

### Effect of SW on the redox status and membrane integrity in tobacco cells

**Fig 5:**
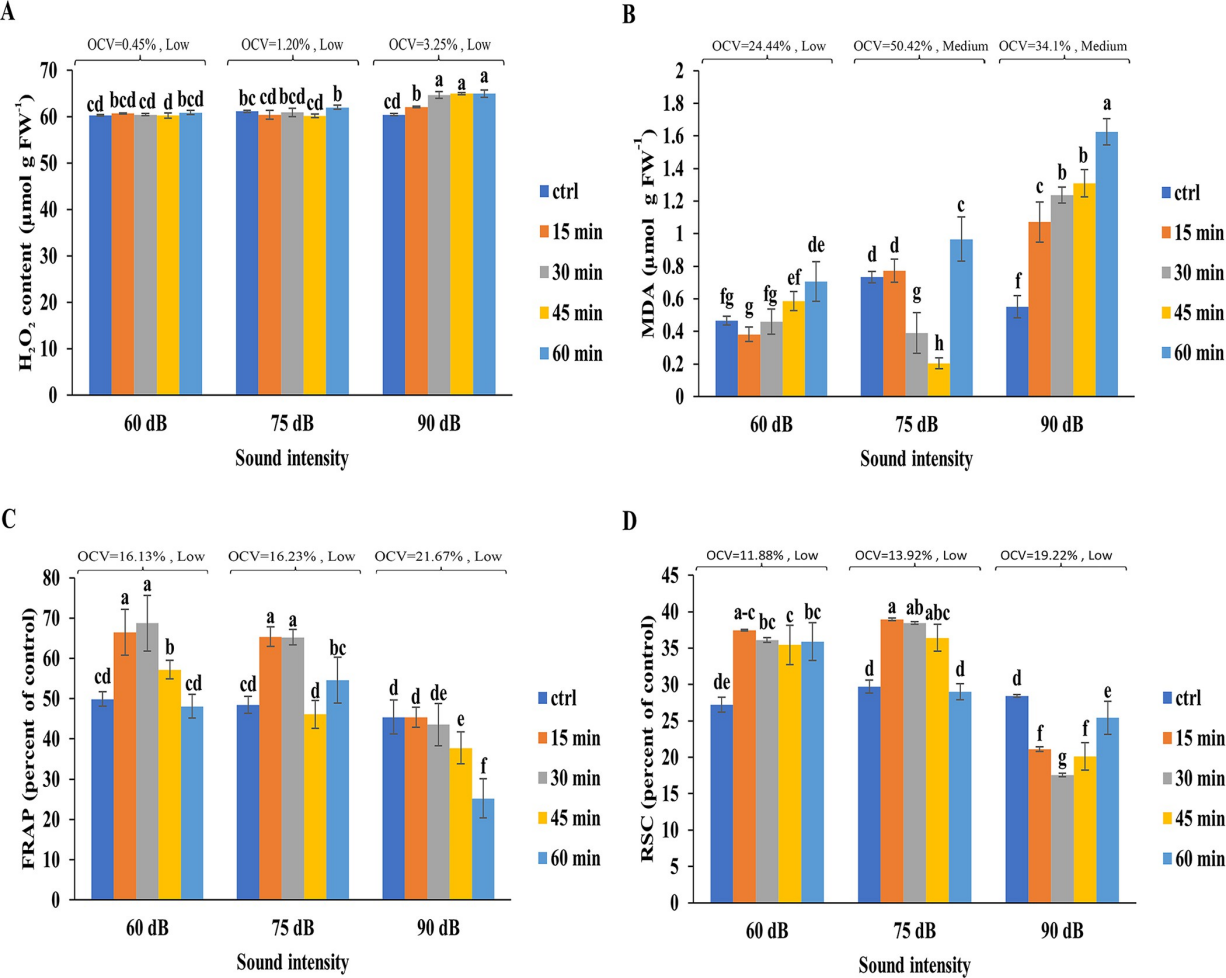
Effect of SW on the Redox status and membrane integrity in tobacco cells. Effect of SW 60, 75, and 90 dB on H_2_O_2_ content **(a)**, MDA **(b)**, antioxidant activity based on the iron-reducing capacity **(c)**, free radical scavenging capacity **(d)** in tobacco cells before and after treatment for 15, 30, 45 and 60 min are shown. Different letters denoted on bars indicate significant differences (Duncan test, *p* ≤ 0.05). OCV was classified as low when it varied from 0.45 to 32.2%, medium from 32.2 to 63.95%, and high from 63.95 to 95.7%.

Treatment of tobacco cells with SW at high-intensity SW 90 dB, in particular for longer than 45 and 60 min, increased the H_2_O_2_ and MDA levels significantly (Fig 5A and 5B). Among different intensities of SW, effects of 75 and 90 dB were more prominent on MDA levels, and the OCVs were 50.42% and 34.1%, respectively, which were considered as medium values. Meanwhile, the OCV of H_2_O_2_ after 60 dB treatment declined to its lowest range, with an OCV of 0.45%. However, the FRAP level that shows the antioxidant activity based on the iron-reducing capacity decreased as a function of SW intensity and exposure time, with the highest level identified at SW 60- and 75 dB (Fig 5C). The RSC was also decreased mainly as a function of SW intensity and treatment time. The highest level was identified at SW 60- and 75 dB (Fig 5D). The OCV for FRAP and RSC were classified as low.

### Effect of SW on the growth characteristics of tobacco cells

**Fig 6:**
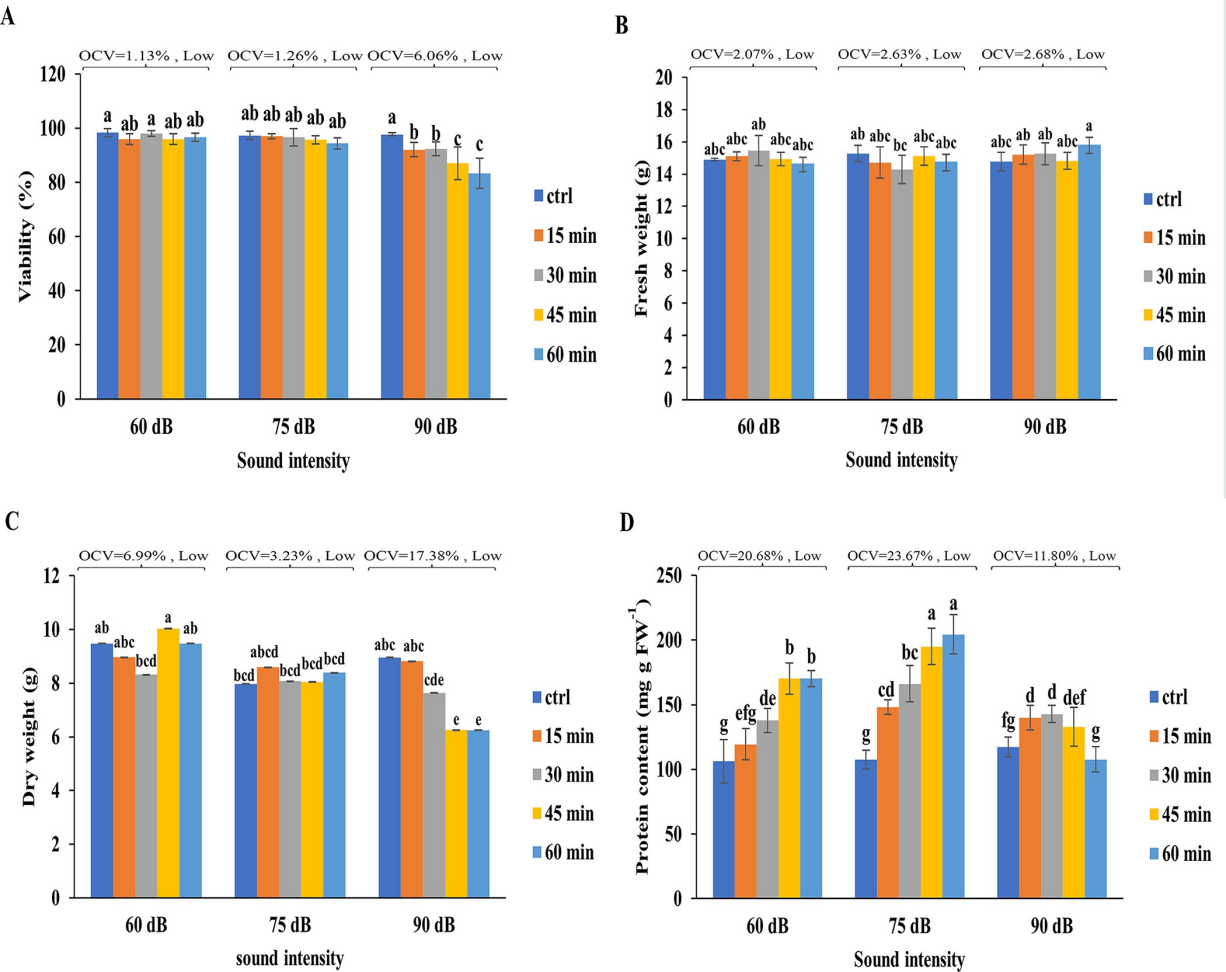
Effect of SW on the growth characteristics of tobacco cells. Growth characteristics of cells before and after treatment with SW 60, 75, and 90 dB for 15, 30, 45, and 60 min, on their **(a)** viability, **(b)** fresh weight, **(c)** dry weight, **(d)** protein after exposure to soundwave are shown. Different letters denoted on bars indicate significant differences (Duncan test, *p* ≤ 0.05). OCV was classified as low when it varied from 0.45 to 32.2%, medium from 32.2 to 63.95%, and high from 63.95 to 95.7%.

Treatment of tobacco cells with SW 90 dB for 45 and 60 min significantly decreased the viability and DW of the exposed cells (Fig 6A–6C). However, the cell exposure to SW with intensities of 60 and 75 dB for 15, 30, 45, and 60 min had no significant effects on their viability (Fig 6A). Likewise, the biomass of the cells was affected by neither SW 60 dB nor 75 dB (Fig 6B and 6C). An increasing trend was observed in the soluble protein content of tobacco cells as a result of their exposure to SW 60- and 75 dB (Fig 6D). The protein content of cells initially increased after 45 min of exposure to SW 90 dB but returned to the control level after 60 min (Fig 6D). The OCVs of FW, DW, viability, and protein content were classified as low.

### Correlations among different parameters after exposure to SW by PCA

**Fig 7:**
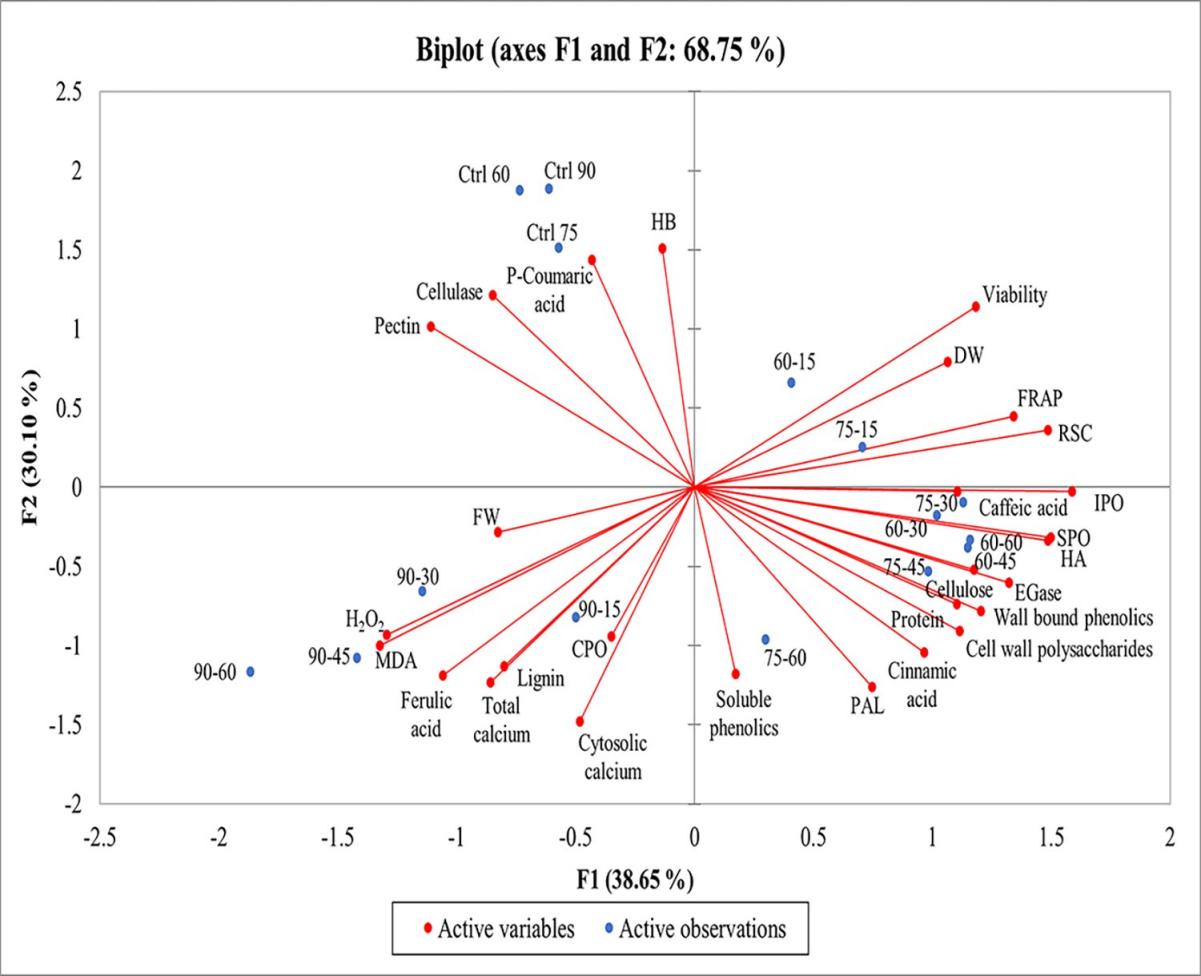
Principal component analysis of physiological parameters under SW treatment.

The potential correlations between studied variables at different SW treatments were analyzed based on Pearson’s correlation coefficient analysis. The results revealed characteristics of principal component 1 (F1) and principal component 2 (F2), representing 38.65% and 30.10% of the total variation with a cumulative percentage of 68.75%, respectively. The PCA results demonstrated the boundary between the SW treatments and the control group was clear which all control groups were on the left upper side of the chart near together, far from treatments. Correlation analysis demonstrated close relationships between high intensity sound treatment (90 dB) and MDA and H_2_O_2_ which were negatively correlated with DW and viability (Fig 7).

The viability and DW were associated with FRAP, RSC, IPO, SPO, protein, HA, HB, cellulose, cell wall polysaccharide, cellulase, EGase, caffeic acid, cinnamic acid, and p-coumaric acid but showed negative correlation with lignin, ferulic acid, CPO, total Ca^2+^, cytosolic calcium, and pectin. Ferulic acid, lignin, and CPO were positively correlated with each other (Fig 7).

## Discussion

Sound waves are pressure waves that have a mechanical influence on plant cells. Mechanical stimuli in plants are either received via the cell wall, cell membrane, and cytoskeleton continuum or through mechano-sensitive small conductance (MscS) and MscS-like (MSL) channels [42].

In the present study, the exposure of tobacco cells to 75- and 90 dB SW increased the total content of cellular Ca^2+^, revealing more Ca^2+^ influx permitted by membrane-localized Ca^2+^ permeable cation channels, possibly MSLs and MCA [2, 43]. This was approved by the high and medium OCV for the total calcium concentration, particularly in cells treated with SW at 75 dB and 90 dB. Exposure of tobacco cells to SW also increased cytosolic Ca^2+^ contents. Intracellular Ca^2+^ is one of the most important players in the plants’ signal transduction pathways and briefly increases upon receiving a stimulus [44].

Cytosolic free Ca^2+^ can bind to the EF-hand motifs of certain proteins, e.g., calmodulin, and activate kinases, and subsequently result in phosphorylation and activation of transcription factors. Upregulation of CREB, a Ca^2+^-regulated transcription factor phosphorylated by calmodulin-dependent kinases has been observed in human mesenchymal stem cells after exposure to sound waves of 1 kHz, 81 dB [45]. Transcriptomic analysis of Arabidopsis upon treatment with SW (500 Hz, 80 dB) has shown up-regulation of some kinases (e.g. MPK11) and transcription factors (MYB77, DREB26, and RAV1). These kinases are regulated by Ca^2+^ which corroborates differential gene expression upon SW treatment [2]. Cytosolic calcium can also bind to EF-hand motifs of respiratory burst oxidase homologs (RBOHs) which produce superoxide anion radicals which are then converted to H_2_O_2_ [46].

Like Ca^2+^, ROS also can lead to the activation of several transcription factors that facilitate differential gene expression [46]. This possibility is further strengthened by SW- mediated (50 Hz, 90 dB) up-regulation of some touch-responsive genes (e.g. TCH2, TCH3, and TCH4) in Arabidopsis [2].

It has been shown that SW, as a mechanical stimulus, activates phospholipase C (PLC) and consequently increases inositol 3-phosphate (IP3) and intracellular Ca^2+^ [47]. Applying continuous sound waves with the intensity of 100 dB and frequency of 800 Hz on *Chrysanthemum* callus cells revealed an increase in cytoplasmic Ca^2+^ concentration and major redistributions of Ca^2+^ occurring between cellular compartments, i.e., nucleus, Golgi apparatus, and vacuole [48]. Treating *Chrysanthemum* callus cells with EGTA, the specific chelating agent, reduced the effects of sound, but when the cells were grown in a Ca^2+^-depleted medium, the stimulatory effect of SW still occurred. This suggested that necessary Ca^2+^ might be supplied by the influx through cell walls [49]. Although the average concentrations of cytosolic Ca^2+^ after exposure to SW were significantly higher than controls, the OCV of internal calcium was classified as low. This may denote that the measurement of cytosolic Ca^2+^ by Fura2-AM has not been sensitive enough. This is consistent with many reports where the accuracy of this method was questioned as the fluorescence and confocal microscopic analysis of some cells by Fura-2 revealed that not only Ca^2+^ was accumulated in the cytosol but also (and especially) within multiple, discrete subcellular compartments i.e., mitochondria and endoplasmic reticulum [50, 51]. In other words, when the distribution of the dye is compartmentalized, accurate measurement of cytosolic Ca^2+^ is unfeasible [50].

Cell wall rigidity is the result of peroxidase-mediated cross-linking of several compounds, such as lignin and phenolic monomers [37]. This process, which is catalyzed particularly by CPO, was associated with a reduction of extensibility of the cells and cell growth slowing down in SW 90 dB treated cells [52]. Mechanosensitive ion channels located in the cell membrane of tobacco cells that are responsible for the uptake of Ca^2+^ could be the target of the applied SW in the current study [8]. The proteins in eukaryotes normally are too delicate, and a minor pressure of about 5–8 pico-Newton suffices to change their conformation and effect on their gaiting [53]. Thus, the effective mN forces imposed hereby SW is justified. Accordingly, imposing the pressure 1,000 times per second on the tobacco cells with NtMCA1 and NtMCA2 Ca^2+^-dependent channels at different pressures correlates with the responses we monitored by means of free Ca^2+^ vitiation and the corresponding subsequent effects on different aspects of cell activities [54]. The richest Ca^2+^-containing component of the cell wall is pectin, which forms a gel-like structure and strengthens the wall [54]. In the present study, the pectin content of SW-exposed tobacco cells was significantly lower than that of controls. This phenomenon was accompanied by a significant increase in EGase activity, suggesting that upon stimulation by SW, the pectic degrading enzyme was activated, and the Ca^2+^ embedded in pectin was released to the cytoplasm. It has been shown that SW- induced changes in the concentration of cytoplasmic Ca^2+^ can activate several Ca^2+^-binding proteins and Ca^2+^-dependent protein kinases (CDPKs), thereby altering the pattern of cell growth and development [55, 56].

The total content of CWP, cellulose, and HA in tobacco cells remarkably increased due to treatment by 60 SW. Likewise, OCVs of these parameters were classified as medium. OCVs for cellulose, HA, and HB were also medium at 75- and 90 dB SW treatments, which shows that exposure to SW affected cell wall components. This can be attributed to the increase of cellulose synthase activity mediated by SW-induced increase of cytosolic Ca^2+^ and the function of certain CDPKs. This is consistence with the recent findings of Xin et al. [57] who uncovered a novel CDPK (CPK32) that regulates cellulose biosynthesis, motility, and bidirectional movement of cellulose synthase complexes.

The increase of cellulose in walls of 60- and 75 dB SW-treated cells indicates lower cellulase activity in these cells. Recent research has shown that there is a direct interaction between pectin and cellulose, so a modified and controlled degradation of pectin is associated with changes in cellulose [58, 59]. The decrease in pectin content of SW-treated cells could be compensated by an increase of firmer components, e.g., cellulose and hemicellulose. The latter associates with cellulose and pectin non-covalently, forming a network that contributes to increasing the load-bearing capacity of cell walls. An increase in hemicellulose content could enhance cell wall stiffening and prevent cell collapse [60].

It was intriguing that exposure of tobacco cells to SW of 60 and 75 dB brought no significant changes in H_2_O_2_ and MDA but increased FRAP and RSC. The increase of radical scavenging and antioxidant capacity of these cells can result from the increase of strong H_2_O_2_ scavengers, including enzymatic, i.e., SPO and IPO, and non-enzymatic ones, phenolic compounds [61]. Thus, considering recent reports on the stimulation of plant cells with SW, where the amounts of reactive oxidative species (ROS) transiently increased [4], more investigation should be conducted. Among ROS, H_2_O_2_ has a relatively long biological lifespan, making it a good indicator to predict cell redox status [62]. It causes significant damage to the plasma membrane [63]. As mentioned, the most promising molecular candidate functioning as a second messenger of SW is Ca^2+^. There are interconnections between ROS and Ca^2+^ signaling in the perception and transmission of environmental signals in plants [64]. It has been shown that the activities of certain ROS-generating enzymes are regulated directly or indirectly by the concentration of Ca^2+^ [65]. Previous studies have indicated that Ca^2+^ plays dual roles in regulating ROS homeostasis [65, 66]. The net Ca^2+^ effects on ROS generation and annihilation appear to be context-sensitive and, even within a given cell, are differentially regulated in local subcellular compartments [67]. Among ROS, H_2_O_2_ is directly used by certain wall-modifying enzymes such as CPO. An increase of the latter in 90 dB SW-treated tobacco cells in the present study resulted in crosslinks between various wall substances i.e., pectin, lignin, and phenolics, thereby limited the growth of the cells and reduced their DW. An increase of peroxidase isoenzymes has been reported in *chrysanthemum* seedlings after treatment with 100 dB, 1,000 Hz SW [68]. Decreases in the weight of roots and the green parts of strawberry has been observed after exposure of the plants to SW of 100 and 105 dB at a constant frequency of 1,000 Hz [15].

The increase of phenolic acids, particularly cinnamic acid, was a consequence of increased PAL activity in SW-treated cells. Increased expression of the PAL gene enhanced activity of this enzyme, and subsequent increase of phenolic compounds and antioxidant capacity have also been observed in red radish, lettuce, and Chinese cabbage after exposure to audible sounds [61, 69]. Interestingly, despite the increase of cinnamic and caffeic acid, no trend of increase was observed in lignin content of 60- and 75 dB treated cells, suggesting that phenolic acids served their connection with pectin to the maintenance of cell viability as evidenced by the increase of total protein and stability of cell biomass. Exposure to SW 90 dB, however, was accompanied by a drastic increase of ROS and membrane lipid peroxidation rate and subsequent reduction of radical scavenging capacity, viability, and DW, while their FW was not changed. The latter might be attributed to the activation of CDPKs upon exposure to SW and an increase of cytoplasmic Ca^2+^. Phosphorylation of cell membrane located aquaporins results in more water uptake and maintenance of the FW of the cells [70].

The PCA analysis reported in the present study could help to better understand the influence of SW on the physiological and biochemical characteristics of tobacco cells. Based on PCA analysis, SW negatively affected tobacco cells at high SW intensity. Moreover, PCA grouping revealed a positive correlation between H_2_O_2_ and MDA levels and 90 dB SW at different exposure durations as indicators of oxidative damage. There was also a positive correlation between SW intensity and total calcium concentration, so the highest Ca^2+^ was observed at the highest SW. The PCA analysis also showed a negative correlation between viability and DW with H_2_O_2_ and MDA, which was consistent with the damaging characteristics of these two parameters. These correlations confirmed again the role of high intensity SW in stimulation of internal Ca^2+^, and activation of downstream changes of redox status, decrease of membrane integrity, stiffening of cell wall, and reduction of biomass.

## Conclusions

Based on the results presented here, the energy of the applied SW was perceived by individual tobacco cells and translated into cellular and metabolic changes mediated by Ca^2+^ signaling. The latter triggered consequence downstream signaling pathways, which altered the redox status of cells. There is likely a discriminating threshold for sound waves, so a 15 dB change in sound intensity from 75 to 90 dB led to significant changes in the biochemical and physiological responses of tobacco cells. In this connection, H_2_O_2_ plays a central role with dual functions. In exposure to SW 60 and 75 dB, the level of H_2_O_2_ was low, functioned as a second messenger, and promoted defense reactions that resulted in the maintenance of membrane integrity and increase of radical scavenging capacity of the cells. On the contrary, treatment with SW 90 dB caused a higher concentration of H_2_O_2_, increased MDA due to oxidative damage incurred on lipid membrane, stiffened walls, and consequently reduced the growth and viability of the cells. Based on the justified correlation between SW pressures and Ca^2+^ level changes in tobacco cells, we are trying to find out the correlation factor between different SW dB and Ca^2+^ changes that acted as secondary messengers triggering various physiological dominos to use it as a manipulative means to control cell activities, growth, immunity and so on.
